# Unveiling the Therapeutic Potential of Systemic Ozone on Skin Wound Repair: Clinical, Histological, and Immunohistochemical Study in Rats

**DOI:** 10.1155/2024/6623114

**Published:** 2024-08-20

**Authors:** Jânderson de Medeiros Cardoso, Edilson Ervolino, Erton Massamitsu Miyasawa, Leticia Helena Theodoro, Luis Eduardo Marques Padovan, Estevão Lopes Pereira, Rafael Scaf de Molon, Valdir Gouveia Garcia

**Affiliations:** ^1^ Department of Implantology Latin American Institute of Dental Research and Teaching (ILAPEO), Curitiba, PR, Brazil; ^2^ Department of Basic Sciences School of Dentistry São Paulo State University (UNESP), Araçatuba, São Paulo, Brazil; ^3^ Department of Diagnostic and Surgery School of Dentistry São Paulo State University (UNESP), Araçatuba, São Paulo, Brazil

**Keywords:** animal experimentation, histology, ozone, rats, wound healing

## Abstract

This study sought to examine the effects of systemic ozone (O_3_) treatment on the healing of skin wounds induced on the dorsal surface of Wistar rats. The skin wounds were created using a 10 mm round punch following the sagittal medial plane in 72 rats. Then, the animals were randomly assigned to four groups, each receiving the following treatments: group C, which did not undergo treatment with the O_3_/O_2_ mixture; group OZ0.3, administered the O_3_/O_2_ mixture at a dose of 0.3 mg/kg; group OZ0.7, given the O_3_/O_2_ mixture at a dose of 0.7 mg/kg; and group OZ1.0, provided with the O_3_/O_2_ mixture at a dose of 1.0 mg/kg. Six animals from each group were euthanized at 7, 14, and 21 days postoperatively. Clinical, histological, histometric, and immunohistochemical (IHC) analyses were accomplished. Data from clinical and histometric assessments revealed that OZ0.7 and OZ1.0 demonstrated more favorable healing, with greater wound contraction observed in the OZ1.0 group at 14 and 21 days. Histologically, the OZ1.0 group exhibited aspects consistent with an accelerated tissue repair process. IHC analysis revealed greater vascular endothelial growth factor (VEGF) immunostaining in the OZ0.7 (7 days) and OZ1.0 (7 and 14 days) groups compared to the C group. Expression of transforming growth factor beta-1 was significantly increased in the OZ0.7 (14 days) and OZ1.0 (7 and 14 days) groups compared to the C group. In conclusion, our data suggest that systemic use of O_3_ enhanced tissue repair in cutaneous wounds in a dose-dependent manner, with concentrations of 1.0 mg/kg providing the most beneficial effects. Furthermore, the results of this study implicate the use of O_3_ for the treatment of skin wounds aiming at improving the healing process over time. Our findings suggest the use of O_3_ as a viable alternative to enhance wound healing and repair.

## 1. Introduction

The wound healing process is a complex series of phases, beginning with hemostasis, followed by the inflammatory, proliferative, and remodeling phases [1]. During hemostasis, a fibrin network is formed, serving as a scaffold to retain blood cells, plasma, and platelets, thereby forming a blood clot. This clot also acts as a source of growth factors and chemokines, recruiting inflammatory cells (ICs) to migrate to the wound site [2–4]. Adhesion molecules initiate their action on endothelial cells, allowing the recruitment and extravasation of neutrophils and macrophages capable of releasing crucial inflammatory mediators that control the healing process, such as interleukins (IL-1, IL-6), tumor necrosis factor-alpha (TNF*α*), platelet-derived growth factor (PDGF), fibroblast growth factor-2 (FGF2), transforming growth factor-beta (TGF*β*), and vascular endothelial growth factor (VEGF) [5–8].

The action of these molecules leads to vascular neoformation (angiogenesis) and the differentiation of forming cells, such as fibroblasts, which proliferate and initiate the deposition of collagen matrix [5–8]. This matrix provides a substrate for the migration of keratinocytes from the wound margins, guiding them at the interface between the clot and the underlying dermis, thus characterizing the proliferative phase. This process culminates in the epithelial closure of the entire wound, referred to as reepithelialization [5–8].

Various critical factors contribute to the unsatisfactory occurrence of the wound healing process, such as vascular insufficiency in the area, systemic disorders like diabetes mellitus, and blood pressure effects, leading to the development of chronic wounds [1]. In response to this challenge, the scientific community has been exploring local or systemic approaches, methods, and treatments aimed at promoting a more regular, uniform, and stable repair process. These encompass photonic therapy, electrical currents, tissue engineering, skin grafting, herbal remedies, stem cells, biomembranes, and ozone therapy.

Ozone is composed of a mixture containing 5% ozone and 95% oxygen that comes in gas or liquid forms (water or oil) [9, 10]. It can be administered either locally, using ozonized water, ozonized oil, or gas, or systemically through methods such as rectal insufflation, intramuscular, subcutaneous, and intraperitoneal injection. The objective is to enhance tissue oxygenation, boost the body's defense system, and exert a lethal impact on microorganisms in areas affected by infection [11–13]. In dentistry, recent publications have focused on studying its mechanism of action and effects on dental caries [14], canal and cavity disinfection [15], gingivitis [16], implant dentistry [12, 17–19], biofilm control/periodontitis [19–23], and jaw osteonecrosis [24]. Moreover, the use of ozone therapy has been applied to manage postextraction wound healing [25–27] and palatal wound healing [28]. In the medical field, ozone has been tested to accelerate the healing of cutaneous wounds [29–32], dermatological diseases, and pain management [11, 12]. Ozone displays a diverse array of effects including antimicrobial, anti-inflammatory, antioxidant, and antihypoxia effects. These properties of ozone make it suitable to treat cutaneous wounds, but their effects on the healing of skin wounds remain to be elucidated.

The literature on ozone therapy reveals conflicting findings, primarily due to the diverse results encountered and the different methodologies employed (administration via, dosages and regime duration). For instance, a recent study has shown that the application of highly ozonated sunflower oil does not improve palatal wounds in a randomized clinical trial [28]. On the other hand, Patel et al. [33] showed significant improvements in the healing of palatal wounds after gingival graft removal. According to a systematic review [34], wounds treated with topical ozone had a greater reduction in wound size. Wounds treated with ozonated liquids also had a shorter time to tissue healing by approximately 1 week [34]. Furthermore, numerous studies have demonstrated positive outcomes for socket wound healing [25, 26], alveolar osteitis [35], and treating dermal and skin wounds [29–32].

Although several studies have investigated the potential effects of ozone in the dermatology field, the literature is still scarce regarding its effects to heal cutaneous wounds. Therefore, this study is aimed at assessing the impacts of systemic ozone (O_3_) therapy and the influence of various dosages of the O_3_/O_2_ mixture on the healing of open wounds induced on the dorsum of rats. The main hypothesis is that O_3_ will enhance tissue repair, and the dosage might impact the biological response. The null hypothesis, on the other hand, is that there will be no added benefits from employing O_3_ in the healing of cutaneous wounds.

## 2. Materials and Methods

### 2.1. Animals

Seventy-two male albino Wistar rats (*Rattus norvegicus*) aged between 3 and 4 months and with an average weight of 335 g, were used in this study. The rats were sourced from the Animal Facility at the School of Dentistry on the Araçatuba UNESP. All animals underwent an acclimatization period for at least 1 week before the start of the experiment. Throughout the entire investigation, they were housed in plastic cages with four animals per cage and maintained in a climate-controlled environment with a temperature of 22 ± 2°C and a 12–12 h light-dark cycle.

During the experimental period, the animals were provided with crushed solid food (Presence Feed, Primor S.A. Mill, São Paulo, SP) and had access to water “ad libitum.” The study received approval from the Animal Ethics Committee of the School of Dentistry in Araçatuba, UNESP (Process 0287-2021), and all procedures adhered to ethical principles of animal experimentation and followed the ARRIVE guidelines for the care and use of laboratory animals [36].

### 2.2. Sample Calculation

The sample size calculation was conducted using the GPower® program, considering an alpha (Type I) and beta (Type II) error of 5% and 80%, respectively, and a medium effect size of ES = 0.25. Based on the number of groups and the experimental time frames, the total required sample size was determined to be 64 animals. To account for potential complications and sample losses, a margin of 15% was included, resulting in a total number of 72 animals.

### 2.3. Surgical Procedures for Wound Creation

For the creation of wounds, animals underwent general anesthesia induced by intramuscular injection of a combination of ketamine hydrochloride (80 mg/kg/bw) and xylazine hydrochloride (10 mg/kg/bw). Then, the dorsal region of the animals was gently shaved, and the area was aseptically prepared with a 10% povidone-iodine topical solution. Subsequently, a single wound was created in the middorsal portion of each animal using a circular punch with an internal diameter of approximately 10 mm, following the animals' midsagittal plane. To standardize the wound, two markings were made on the outer surface of the punch, representing 50% of its diameter. Next, the skin in the targeted area of the animal was folded along the midsagittal plane and carefully supported on the surgical table. The punch was positioned with the markings aligned at the skin edge, and with the assistance of a surgical hammer, a gentle and single strike was executed, allowing the release of the skin fragment. After tissue release, a circular wound with regular edges was obtained for all animals.

### 2.4. Randomization and Treatment Assignment

The animals were randomly assigned using a table generated by the website Randomization.com (http://www.randomization.com/) to four groups that were divided according to the type of treatment, as follows: Control group (C): The animals received no systemic treatment. OZ0.3 group: The animals received an intraperitoneal dose of 0.3 mg/kg/bw of O_3_, with a concentration of 15 *μ*g/ml of O_3_ released by the generator; OZ0.7 group: The animals received an intraperitoneal dose of 0.7 mg/kg/bw of O_3_, with a concentration of 35 *μ*g/ml of O3 released by the generator; and OZ1.0 group: The animals received an intraperitoneal dose of 1.0 mg/kg/bw of O_3_, with a concentration of 50 *μ*g/ml of O_3_ released by the generator. The dosage of ozone was based on previous published studies [37–39].

The ozone generator Philozon Medplus V (Santa Catarina, Brazil) was employed, featuring the following specifications: a safety valve with a vacuum system, automatic regulation of oxygen flow, automatic internal catalyzer, and adjustable flow of oxygen concentrations ranging from 5 to 60 *μ*g/ml. These concentrations were set and displayed on the equipment panel. A single examiner performed the intraperitoneal injections.

### 2.5. Sample Collection and Histological Processing

Six animals from each group underwent euthanasia through an overdose of anesthetic (150 mg/kg, Thiopental®) after 7, 14, and 21 days following the operative procedures and treatments. Soft tissue specimens from the dorsal region encompassing the entire extent of the surgical wound were removed using a Bard-Parker scalpel with a 15C blade and blunt-tipped straight scissors. Care was taken to include a safety margin around the entire wound and cover the entire extent of the subcutaneous connective tissue in the area. The anatomical specimens obtained were labeled, placed in plastic cassettes, and immersed in a 4% formaldehyde solution in 0.1 M phosphate buffer (pH 7.4), remaining for a minimum period of 48 h. After this period, they were directed to the histological processing.

The removed specimens underwent routine laboratory procedures (washed for 24 h in running water, dehydrated in increasing concentrations of ethanol, cleared in xylene, and embedded in paraffin blocks). Semiserial sections with a thickness of 5 *μ*m were made, with sections stained using the hematoxylin and eosin (H&E), and also submitted to immunohistochemical (IHC) analysis.

### 2.6. Clinical Assessment of Wounds

All wounds from each group were clinically evaluated by an experienced and blinded examiner (JMC). The following clinical conditions were observed: wound contraction, presence of visible blood vessels in the wound area, presence and characteristics of crust, edema, bleeding, and signs of inflammation and infection.

### 2.7. Descriptive Histology and Histometric Evaluation

Descriptive histological and histometric analyses from each group and time points were assessed by an expert histologist (EE) blinded to the experimental groups. Photographs of all wounds from each animal and group were taken at all experimental time points (0 h, 7, 14, and 21 days). Each animal was positioned in a ventral decubitus on a surgical table, ensuring that the wound remained as parallel as possible to the ground. A digital camera (Canon 60D, Japan) with a Macro 100 lens (Canon 60D, Japan) fixed on a stand was used to maintain a standardized distance between the wound surface and the camera lens. The images of each animal group were then processed using publicly available image processing software, Image J® (National Institutes of Health, USA). The wound area measurement was conducted by outlining the entire wound perimeter with the mouse cursor on the computer screen, and the obtained measurement was expressed in millimeters (mm).

For the histometric analysis, the characteristics of the epithelium were evaluated to quantitate the area occupied by epithelial tissue (AET) in the surgical wound. Subsequently, the surface occupied by the epithelial tissue (SET) was assessed. In the connective tissue in the central area of the wound, the amount of ICs expressed in percentage of the total area and the area occupied by collagen fibers (ACF) were quantitated. These measurements were performed by two experienced examiners (EMM and JMC) blinded to the experimental groups.

### 2.8. IHC Assessment

Tissue sections underwent the indirect immunoperoxidase method for the identification of VEGF and TGF*β*-1 proteins. Antigen retrieval was achieved by immersing histological slides in a specific buffer (Diva decloaker®, Biocare Medical, Concord, CA, USA) in a pressurized chamber at 95°C for 20 min. Then, the histological slides were washed in 0.1 M phosphate-buffered saline (pH 7.4) and were immersed in 3% hydrogen peroxide (H_2_O_2_) for 1 h and in 1% bovine serum albumin. The slides were incubated with anti-VEGF anti-TGF*β*-1 antibodies for 24 h. The revelation was performed using 3,3′-diaminobenzidine tetrahydrochloride (DAB chromogen Kit®, Dako Laboratories, CA, USA). Counterstaining was carried out with the Harris hematoxylin, dehydrated in ethanol, cleared in xylene, and covered with mounting medium and glass coverslips. As a negative control, the primary antibody was omitted, as previously described [40]. A semiquantitative analysis respected a set of scoring criteria, which were as follows: Score 0 represented a null immunostaining pattern, indicating the complete absence of immunoreactive cells (IR). Score 1 indicated a low immunostaining pattern, characterized by approximately 1/4 of IR cells. Score 2 denoted a moderate pattern of immunostaining, with approximately 1/2 of IR cells. Finally, Score 3 represented a high pattern of immunostaining, with approximately 3/4 of IR cells, as described [41].

### 2.9. Statistical Analysis

For the statistical analysis of clinical wound data, the IBM program (International Business Machines Corp., Armonk, New York, USA) was utilized. The Kolmogorov–Smirnov and Shapiro–Wilk tests were employed to assess data normality and sample distribution. Subsequently, a one-way analysis of variance (ANOVA) was applied, followed by the Games–Howell posttest for multiple parametric comparisons in the presence of heterogeneous variances, with a significance level set at 5% (*p* < 0.05).

## 3. Results

### 3.1. Clinical Analysis

**Figure 1:**
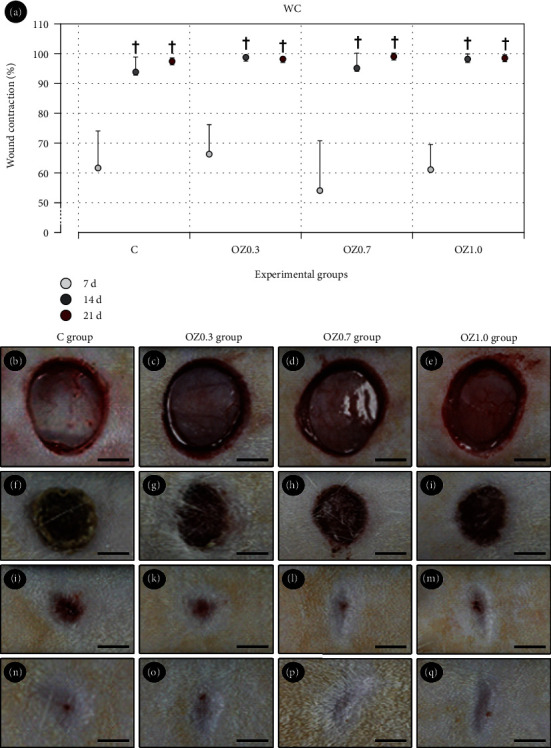
Clinical aspect of surgically created wounds on the back of rats in different groups and experimental periods. (a) Graph illustrating the wound contraction (WC) in groups C, OZ0.3, OZ0.7, and OZ1.0 at 0, 7, 14, and 21 days postoperatively. (b) Photographs highlight the clinical characteristics of the surgically created wounds in groups C (b, f, j, n), OZ0.3 (c, g, k, o), OZ0.7 (d, h, l, p), and OZ1.0 (e, i, m, q) at 0 (b–e), 7 (f–i), 14 (j–m), 21 days postoperative. Symbols: †, statistically significant difference compared to 7 days within the same group. Scale bars: 5 mm.

The wound evaluation followed a standardized approach, documenting all clinical evidence of repair, including contraction, visible blood vessels, presence and characteristics of crust, edema, bleeding, inflammation, and signs of infection (Figure 1). Immediately after creating the cutaneous wounds, all wounds showed sharp edges and a regular size, close to the diameter of the circular punch, demonstrating the effectiveness of the method for their creation. Capillaries were observed in the dermis, and there were no signs of significant bleeding, infection, or inflammation within the wounds (Figure 1). At 7 days postoperatively, wounds in the C group exhibited the presence of a dry crust, with signs of partial displacement at the wound margin along its entire length. Wounds in the OZ0.3, OZ0.7, and OZ1.0 groups showed a wet crust, with some areas beginning to detach at the margin, which was similar in clinical characteristics in these groups. By day 14 postoperatively, all wounds in all groups showed complete displacement of the crust; however, those in the C group exhibited a lower degree of contraction compared to the other groups. In the comparative analysis among wounds in all groups, those in the OZ0.7 and OZ1.0 groups demonstrated a more advanced state of healing, with greater contraction and a smaller area of redness in their central portion. At 21 days, all wounds showed a total absence of the crust, but wounds in the C and OZ0.3 groups still displayed a slightly reddish area in their central portion compared to those in the OZ0.7 and OZ1.0 groups, which demonstrated complete closure of the wound with scar formation. There were no wounds presenting with undesirable side effects such as bleeding or infection throughout the course of the study.

Regarding the degree of wound contraction, no statistically significant difference was detected among the experimental groups (Figure 1). At 14 and 21 days, the degree of contraction of surgical wounds was greater than at 7 days in all experimental groups (Figure 1).

### 3.2. Descriptive Histological Analysis

**Figure 2:**
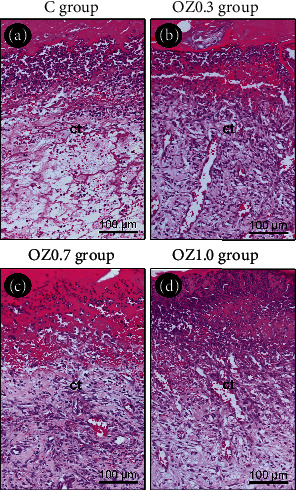
Histological aspect of the skin at 7 days postoperative in the different experimental groups. Photomicrographs highlight the histological characteristics of the connective tissue located at the center of the surgical wound in groups (a) C, (b) OZ0.3, (c) OZ0.7, and (d) OZ1.0 at 7 days postoperative. Abbreviations and symbols: ct, connective tissue. Original magnification: 200x. Scale bars: 100 *μ*m. Staining: hematoxylin and eosin (H&E).

At 7 days postoperatively, the surgical wounds exhibited stratified squamous epithelium proliferating from their edges towards the center. A thin crust was present on the surface of the wound. The connective tissue underlying the epithelium/crust was intensely vascularized, with a large number of ICs and some fibroblasts, supported by a delicate network of collagen fibers (Figure 2).

**Figure 3:**
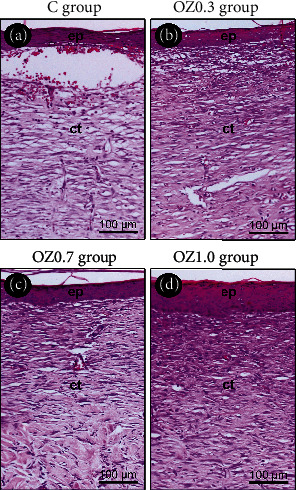
Histological aspect of the skin at 14 days postoperative in the different experimental groups. Photomicrographs highlight the histological characteristics of epithelial and the connective tissue located at the center of the surgical wound in groups (a) C, (b) OZ0.3, (c) OZ0.7, and (d) OZ1.0 at 14 days postoperative. Abbreviations and symbols: ct, connective tissue; ep, epithelial tissue. Original magnification: 200x. Scale bars: 100 *μ*m. Staining: hematoxylin and eosin (H&E).

By day 14 postoperatively, the epithelial tissue covered a large part of the wound. Remnants of the crust were still present in some specimens. The underlying connective tissue was intensely vascularized, with some ICs and abundant fibroblasts, supported by a moderate amount of collagen fibers (Figure 3).

**Figure 4:**
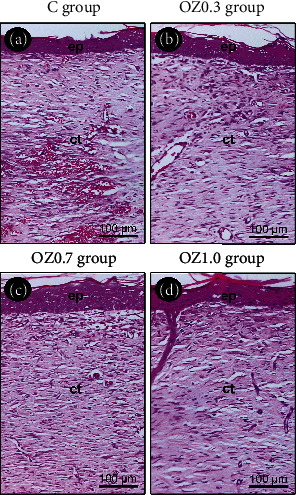
Histological aspect of the skin at 21 days postoperative in the different experimental groups. Photomicrographs highlight the histological characteristics of epithelial and the connective tissue located at the center of the surgical wound in groups (a) C, (b) OZ0.3, (c) OZ0.7, and (d) OZ1.0 at 21 days postoperative. Abbreviations and symbols: ct, connective tissue; ep, epithelial tissue. Original magnification: 200x. Scale bars: 100 *μ*m. Staining: hematoxylin and eosin.

At 21 days postoperatively, the epithelial tissue covered the entire surgical wound. The underlying connective tissue still showed significant vascularity, albeit with few ICs and numerous fibroblasts associated with a large amount of collagen fibers. At this stage, the initiation of the reorganization of cutaneous appendages, such as hair and glands, was observed (Figure 4).

When comparing groups within their respective experimental periods, it was noted that histological characteristics were very similar; however, the OZ1.0 group exhibited some histological aspects consistent with a more accelerated tissue repair process, such as the thickness and cellular pattern of the epidermis and the amount of collagen fibers in the dermis.

### 3.3. Histometric Analysis

**Figure 5:**
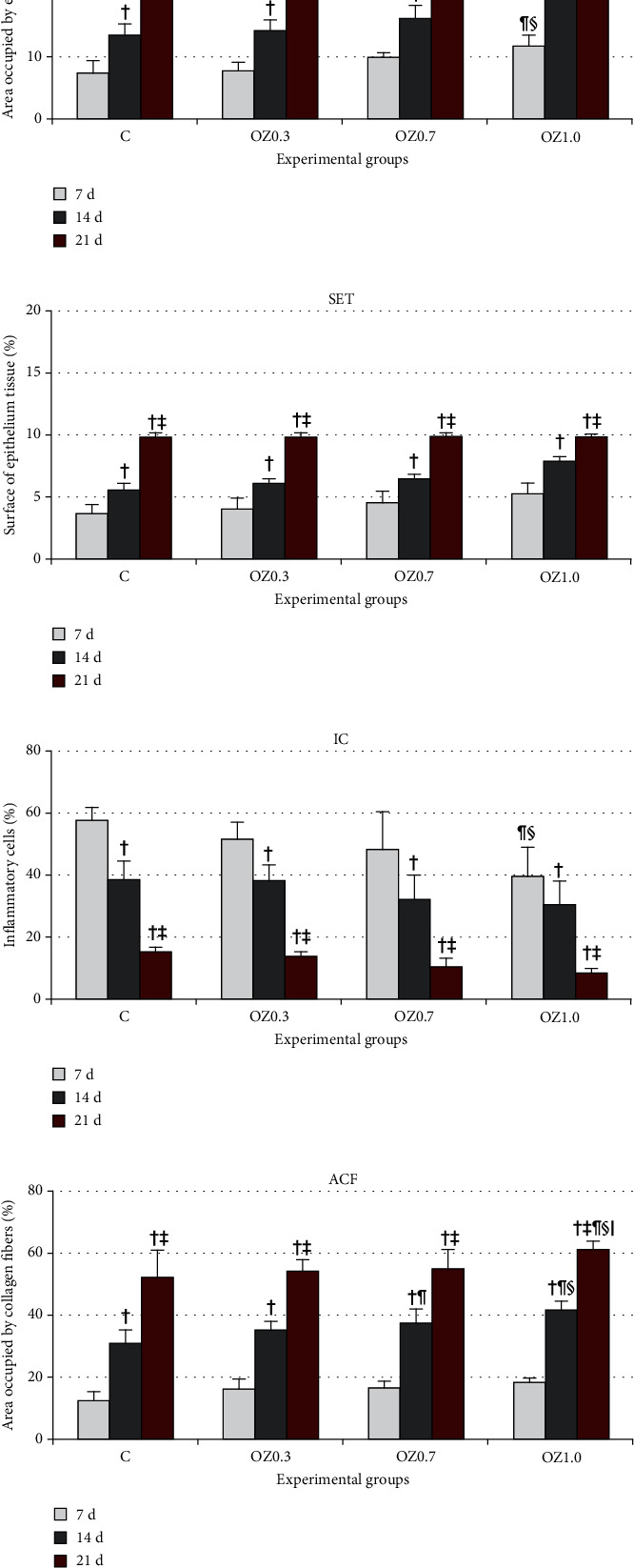
(a) Graphics illustrating the area occupied by epithelial tissue (AET), (b) surface of epithelial tissue (SET), (c) quantitation of inflammatory cells (IC), and (d) area occupied by collagen fibers (ACF) in groups C, OZ0.3, OZ0.7, and OZ1.0 at 7, 14, and 21 days postoperatively. Symbols: †, statistically significant difference compared to 7 days within the same group; ‡, statistically significant difference compared to 14 days within the same group; ¶, statistically significant difference compared to group C during the same period; §, statistically significant difference compared to group OZ0.3 during the same period; |, statistically significant difference compared to group OZ0.7 during the same period.

Initially, the degree of reliability between the evaluators was assessed, obtaining an intraclass correlation coefficient (ICC) of 0.960. The intergroup analysis showed a statistically significant increase in the percentage of AET for the OZ0.7 and OZ1.0 groups compared to the C and OZ0.3 groups (Figure 5(a)). The OZ1.0 group showed the most promising effects, meaning that the O_3_ effects are dose-dependent (Figure 5(a)). Regarding the quantitation of SET, there were no differences among the experimental groups, but the epithelial coverage increased over time for all groups (Figure 5(b)). Furthermore, the quantification of ICs showed a significant increase in the initial periods of evaluation for all groups. The percentage of ICs decreased for all groups in the later periods of evaluation especially at 21 days. There were no differences regarding the percentage of ICs among groups (Figure 5(c)). The ACF was also evaluated. Our findings showed that the OZ1.0 group showed the most promising results regarding the amount of collagen fibers compared to the other groups (Figure 5(d)).

### 3.4. IHC Analysis

**Figure 6:**
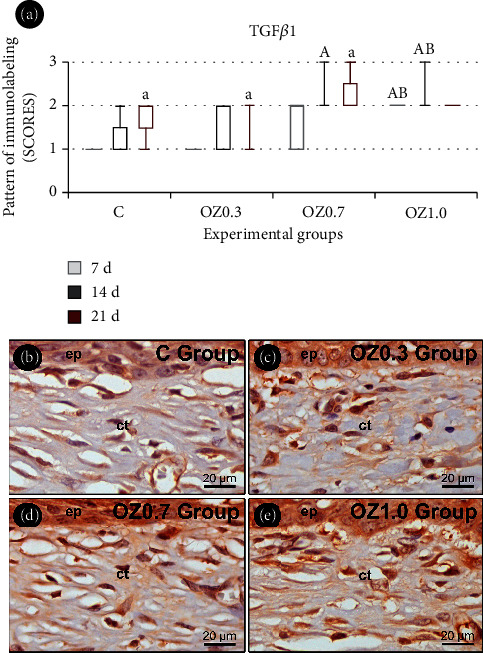
Immunolabeling for TGF*β*1 in the skin in different experimental groups. (a) Graph illustrating the pattern of immunolabeling for TGF*β*1 in the surgical wound in groups C, OZ0.3, OZ0.7, and OZ1.0 at 7, 14, and 21 days postoperatively. Photomicrographs highlighting the pattern of immunolabeling for TGF*β*1 at the base of the epithelial tissue and in the connective tissue located at the center of the surgical wound in groups (b) C, (c) OZ0.3, (d) OZ0.7, and (e) OZ1.0 at 14 days postoperatively. Abbreviations and symbols: et, epithelial tissue; ct, connective tissue; a, statistically significant difference compared to 7 days within the same group; A, statistically significant difference compared to group C during the same period; B, statistically significant difference compared to group OZ0.3 during the same period. Original magnification: 1000x. Scale bars: 20 *μ*m. Counterstaining: Harris hematoxylin.

The IHC analysis of TGF*β*1 revealed that in groups C, OZ0.3, and OZ0.7 at 21 days, there was higher immunolabeling compared to the 7-day period in the same group. In the OZ0.7 group at 14 days and the OZ1.0 group at 7 and 14 days, there was higher immunolabeling than in the C group during the same period. Additionally, in the OZ1.0 group at 7 and 14 days, there was higher immunolabeling than in the OZ0.3 group during the same period (Figure 6).

**Figure 7:**
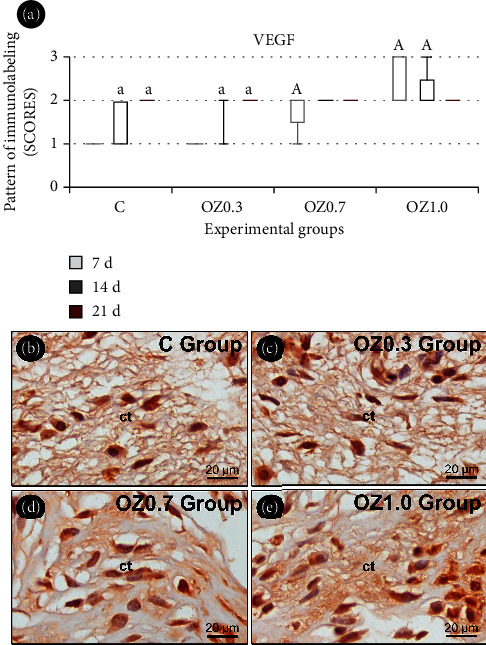
Immunolabeling for VEGF in the skin in different experimental groups. (a) Graph illustrating the pattern of immunolabeling for VEGF in the surgical wound in groups C, OZ0.3, OZ0.7, and OZ1.0 at 7, 14, and 21 days postoperatively. Photomicrographs highlighting the pattern of immunolabeling for VEGF at the base of the epithelial tissue and in the connective tissue located at the center of the surgical wound in group (b) C, (c) OZ0.3, (d) OZ0.7, and (e) OZ1.0 at 14 days postoperatively. Abbreviations and symbols: ct, connective tissue; a, statistically significant difference compared to 7 days within the same group; A, statistically significant difference compared to Group C during the same period. Original magnification: 1000x. Scale bars: 20 *μ*m. Counterstaining: Harris hematoxylin.

The IHC analysis of VEGF revealed higher immunolabeling in groups C and OZ0.3 at 14 and 21 days, compared to the 7-day period in the same group. Additionally, there was higher immunolabeling of VEGF in the OZ0.7 group at 7 days and the OZ1.0 group at 7 and 14 days, compared to the C group during the same period (Figure 7).

## 4. Discussion

The present study assessed, from clinical, histological, histometric, and IHC perspectives, the effects of systemically administered ozone and the influence of different dosages on the wound repair in surgical defects created in rats. The results of this study suggested a beneficial action of the O_3_/O_2_ mixture in stimulating wound repair in a dose-dependent manner, confirming our study hypothesis. From a clinical perspective, our data reveal that wounds treated with ozone displayed a dry crust, exhibiting greater contraction than the control wounds across all experimental time points, particularly notable in the OZ0.7 and OZ1.0 groups at 14 and 21 days. These clinical observations find support in histometric results, where wounds treated with the highest ozone dosage at 14 and 21 days demonstrated more significant contraction compared to the other groups.

The most commonly used method of ozone application is topical, whether using ozonized water, ozonized oil, gel formulations, or gas [33, 34, 42, 43]. Systemic administration is achieved through intramuscular injection [44], subcutaneous, and intravenous routes [45], with rectal insufflation being the preferred method in various studies [46]. However, intraperitoneal administration has been employed in the medical field for treating various pathologies [29, 47, 48]. Therefore, we choose to treat the animals with systemic administration of ozone to stimulate biological events in skin tissue repair.

Wound contraction is determined by specialized fibroblasts known as myofibroblasts, containing actin filaments, myosin, and smooth muscle actin (*α*-SMA) in their cytoplasm [49, 50]. Myofibroblasts possess twice the contractile strength of normal fibroblasts [51]. The differentiation of fibroblasts into myofibroblasts in the lesion area is primarily attributed to the activity of TGF-*β*1 [52], which is released in large quantities by platelets during bleeding from the surgical procedure. In the wound repair process, TGF-*β*1 is generated by leukocytes, macrophages, fibroblasts, and keratinocytes, influencing these cells to stimulate the infiltration of ICs, fibroplasia, matrix deposition, and angiogenesis in the area. The results of our study demonstrated a higher staining of TGF-*β*1 in both OZ0.7 (14 days) and OZ1.0 (7 and 14 days) groups compared to the C group. These findings suggest that the employed O_3_O_2_ in these groups was suitable to promote greater cellular differentiation, in a dosage-dependent manner.

The disruption of the vascular network during tissue injury and the high oxygen consumption by metabolically active cells in the area create an environment with reduced oxygen levels (hypoxia). In this hypoxic environment, important cytokines are produced, such as TNF-*α*, PDGF, TGF-*β*, and VEGF, which are crucial promoters of cell proliferation, migration, chemotaxis, and angiogenesis in wound healing [53, 54]. Our data revealed that the immunolabeling of VEGF was higher in the wounds of the OZ0.7 (7 days) and OZ1.0 (7 and 14 days) groups compared to the C group. This finding indicates the ability of the O_3_/O_2_ mixture used in these groups to promote greater angiogenesis in the injured area, resulting in benefits in tissue repair, especially in the early stages of the healing process. Studies have shown an increase in VEGF in the early stages of repair [55], paralleling observations from another preclinical study that observed an increase in VEGF and TGF-*β*1 between 5 and 7 days after topical application of ozonized oil in cutaneous wounds surgically created in the guinea pig [42].

The histological findings of this study revealed that the histological characteristics of the groups treated with O_3_ were very similar to each other. However, the OZ1.0 group showed some histological aspects consistent with a faster tissue repair process, such as the thickness and pattern of cellularity in the epidermis and the quantity of collagen fibers in the dermis. The area occupied by the epithelial tissue (AET) in the OZ0.7 (21 days) and OZ1.0 groups was increased when compared to the C group. This observation suggested that O_3_ has positive effects on cell differentiation and epithelial migration. Additionally, the area in the connective tissue occupied by collagen fibers was larger in the OZ0.7 group (14 days) and the OZ1.0 group (14 and 21 days) compared to the C group. The higher ozone dosage was capable of reducing the inflammatory infiltrate and activating collagen formation, resulting in differentiated epithelial migration. Our findings align with other studies demonstrating that ozone therapy induces biological effects at doses ranging between 20 and 50 *μ*g/ml [56, 57].

Recent studies have developed membranes loaded with curcumin, chitosan, and sodium alginate using microwave technology to enhance wound-healing processes [58–60]. The findings of these studies indicated a notable increase in reepithelialization (75 ± 2.3%) within a 14-day period following application compared to conventional gauze and other treatments. Additionally, there was a notable enhancement in collagen deposition and stratum corneum formation [58]. Similarly, Rostami et al. [59] demonstrated that a biodegradable film composed of chitosan and nano selenium accelerated full-thickness excisional wound healing, promoting cellular infiltration and neovascularization in rat skin. Finally, Wang et al. [60] observed significantly expedited wound healing in animals treated with chitosan and polyethylene glycol hydrogel membranes containing curcumin compared to a control group. This was evidenced by a notable reepithelization rate of 87.26% and reduced wound size. These findings suggest a potential opportunity to combine curcumin-loaded membranes with ozone therapy as an adjuvant to enhance clinical efficacy. Nevertheless, further preclinical studies are required to investigate the synergistic effects of these approaches.

While our study methodology does not allow inferences regarding the mechanisms of action involved in this favorable biological response, it is known that O_3_ reacts rapidly with tissue fluids such as water and polyunsaturated fatty acids, resulting in the formation of ozonized lipid products and H_2_O_2_ [61]. In addition to the formation of the important free radical, when in contact with blood, O_3_ decomposes, giving rise to other reactive oxygen species (ROS) such as superoxide anion radical (O_2_-), hydroxyl radical (OH-), and nitric oxide (NO), which, in low concentrations, act as vasodilators and stimulate important endogenous growth factors [62]. Studies have also demonstrated that O_3_, upon reaching the cell membrane, forms ROS that activate the nuclear factor kappa-B (NF-*κ*B) pathways, crucial signaling proteins for the cellular nucleus, consequently promoting the activation of intracellular pro and anti-inflammatory cytokine pathways [63].

It is important to bear in mind that our study has some limitations. First, we do not evaluate the toxicity of systemic administration of ozone. Secondly, systemic administration of ozone does not guarantee the same concentration at the local wound. Therefore, local treatment with ozone should be implemented in future studies. Finally, there is no description about the biological mechanisms by which ozone accelerates wound healing. Nevertheless, further research, particularly controlled randomized clinical studies with a substantial sample size, are warranted for the development of clinical usage protocols.

In conclusion, our findings suggest that the systemic use of O_3_ enhanced tissue healing of cutaneous wounds. The dosage plays a significant role in the biological response in a dose-dependent manner. Furthermore, the results of this study implicate the use of O_3_ for the treatment of skin wounds aiming at improving the healing process over time. Our findings suggest the use of O3 as a viable alternative to enhance wound healing and repair. Future research is indicated in the field and might be expanded to use O_3_ for treating palatal wounds after removal of connective tissue graft for root covering procedures, after tooth extraction to improve soft tissue healing and also to manage mucositis and peri-implantitis.

## Data Availability

The data used to support the findings of this study are available from the corresponding author upon request.
